# Long-Zhi Decoction Medicated Serum Promotes Angiogenesis in Human Umbilical Vein Endothelial Cells Based on Autophagy

**DOI:** 10.1155/2018/6857398

**Published:** 2018-05-07

**Authors:** Quan He, Qihua Liu, Yongbin Chen, Jiaquan Meng, Ling Zou

**Affiliations:** Department of Traditional Chinese Medicine, First Affiliated Hospital of Guangxi Medical University, Nanning, China

## Abstract

Ischemic stroke (IS) is a fatal subtype of stroke that lacks effective treatments. Angiogenesis following IS is an effective response that mediates brain recovery and repair. Our previous study demonstrated that long-zhi decoction (LZD), a Chinese herbal formula, promoted angiogenesis in rats of IS model. To further investigate the association between the proangiogenic mechanism of an LZD-medicated serum and cellular autophagy, we evaluated its promotional effect on angiogenesis in human umbilical vein endothelial cells (HUVECs) in vitro. We used HUVECs subjected to H_2_O_2_ to induce injury and observed the effects of the LZD-medicated serum treatment. Cell-based assays included proliferation, migration, and tube formation. To assess the extent of autophagy, transmission electron microscopy was used to measure the number of autophagosomes. Immunofluorescence and Western blotting were performed to evaluate the autophagy-related protein of LC3-II and Beclin-1. The LZD-medicated serum promoted proliferation, migration, and tube formation in HUVECs. The LZD-medicated serum also increased the autophagosomes and the autophagic protein expressions of LC3-II and Beclin-1. The proangiogenic and autophagic activity of LZD provides new cogitations to its clinical application and may lead to potential drug development for treating various vascular diseases, especially in the elderly, in the future.

## 1. Introduction

Ischemic stroke (IS) is a transient episode of a neurological dysfunction caused by the loss of blood flow to the brain with acute infarction, and it has a high morbidity and mortality in the world [1–3]. IS can lead to neurological impairment, brain tissue ischemia, hypoxia, and a serious clinical complication [4]. Therapeutic angiogenesis may enhance the supply of oxygen and nutrients to the affected tissue, thus making it beneficial to the recovery of IS, and extensive evidence suggests that it may be a promising antistroke therapeutic strategy [5]. Recent research has shown that a new vessel formation after IS not only replenishes blood flow to the ischemic area of the brain but also improves neurological functions in both clinical trials and animal models [6, 7]. Moreover, angiogenesis and cell autophagy have a high degree of correlation, and the link between them has received much attention in the current literature [8, 9].

Autophagy (or cellular “self-eating”) plays a pivotal role in various aspects of cell physiology, but flaws in this process are associated with numerous pathological conditions [10]. Autophagy is widespread in eukaryotes, is constitutively at a low level, and is a highly evolutionarily conserved process essential for protecting cellular survival, homeostasis, and integrity [11]. However, different stress conditions involving starvation or organelle deterioration changing nutrient conditions, hypoxia, and pathogen infection result in the upregulation of this process [12]. More importantly, abnormal autophagy is associated with human pathologies, such as cardiovascular, diabetes, neurodegeneration, cancer, pulmonary, and macular degeneration. Cell autophagy plays an important role in a variety of clinical diseases, but the exact mechanism remains unclear [13–15]. Therefore, ongoing work on this topic is necessary to further determine the cellular factors regulating this inherently complex process.

Long-zhi decoction (LZD), a clinical Chinese herbal formula, consists of nine herbs: Huangqi (Radix Astragali seu Hedysari), Shuizhi (Whitmania Pigra Whitman), Chi Shao (Radix Paeoniae Rubra), Niuxi (Achyranthes Bidentata), Danggui (Radix Angelica Sinensis), Taoren (Semenpersicae), Dilong (Pheretima), Honghua (Flos Carthami), and Chuanxiong (Rhizoma Ligustici Chuanxiong). Our research team had already found that LZD could significantly accelerate the rate of capillary formation and promote the recovery of neurological function in an acute ischemic-damaged mouse model compared with the control. However, the mechanisms of this drug for stroke treatment are still unclear. In the present study, we aim to observe the angiogenesis effects and the autophagy changes in LZD on human umbilical vein endothelial cells (HUVECs).

## 2. Materials and Methods

### 2.1. Ethics Statement

All processes in the present research were allowed in accordance with the National Institute of Health Guide and Use of Laboratory Animals, which was also approved by the care of Experimental Animals Committee of the Guangxi Medical University (Nanning, China).

### 2.2. Cell Culture and Chemicals

HUVECs were purchased from the American Type Culture Collection (ATCC®, Manassas, VA, USA). The cell line was cultured in Dulbecco's Modified Eagle's Medium (DMEM) (Invitrogen, Carlsbad, CA, USA) containing 10% fetal bovine serum (GIBCO®, Gaithersburg, MD, USA), 100 IU/mL penicillin, and 100 *μ*g/mL streptomycin in humidified 5% CO_2_ at 37°C. HUVECs at the early passages were used in all the experiments and cultured in gelatin-coated plates with a complete growth medium. For transfection, the cells were grown up to 90% confluence and intervened with H_2_O_2_ using a concentration of 400 *μ*mol/L (AR, Nanning Wilking Biological Technology Co., Ltd., China) before incubation with DMEM for 4 h to establish the base model for the entire experiment. At the completion of the experiments, the cells were washed with precooling phosphate-buffered saline (PBS) for the subsequent experiments.

### 2.3. Preparation of the LZD-Medicated Serum

The LZD-medicated serum was manufactured according to an elapsed report [16, 17]. Twenty Sprague-Dawley rats were randomly divided into the LZD group (*n* = 10) and the control group (*n* = 10). The rats were housed in a 20°C–25°C air-conditioned room with a 12 h light-dark cycle and provided with a standard diet with free access to tap water. The rats in the LZD group underwent intragastric administration of LZD (7.575 g/kg) two times a day for five days. The rats in the blank serum group received intragastric administration of physiological saline twice a day for five days. The volume of intragastric administration of the two groups was consistent. At 1 h after the final administration, the rats were anesthetized using chloral hydrate intraperitoneally and then sacrificed by an intraperitoneal injection of an overdose of chloral hydrate. The rats' blood samples (10 ml) were collected from the abdominal aorta and centrifuged at 3000 ×g at 4°C for 15 min. The serum was isolated and stored at −80°C until further analysis.

### 2.4. Cell Proliferation Assay

The cell suspension of HUVECs was inoculated into a 96-well plate at a density of 5 × 10^3^ cells/mL. The HUVECs were divided into four groups: normal control group, blank serum group, H_2_O_2_ group, and H_2_O_2_ with LZD-medicated serum group. Following treatment, cell proliferation was evaluated using the Cell Counting Kit-8 (CCK8) reagent (Dojindo Laboratories, Kumamoto, Japan). According to the manufacturer's protocol (Nanning Wilking Biological Technology Co., Ltd., China), the absorbance values were read at 450 nm using a plate reader (Thermo Scientific, Watertown, USA).

### 2.5. Wound Scratch Test

Wound scratch test was applied to investigate the migration capability of HUVECs after each group of drug intervention. HUVECs were seeded in 24-well plates and fed with low-glucose DMEM containing 10% FBS. After the cells were grown to approximately 100%, the surface-adherent cells were wounded with a pipette tip. Then, the cells that fell off were flushed with PBS to wash away the floating cells and cellular debris and then cultured with low-glucose DMEM containing intervention drugs. The initial wounding and the migration of HUVECs in the scratched area were observed under an inverted microscope for 24 h. Three different fields from each sample were photographed and quantitatively analyzed by counting the cell number between the borderlines.

### 2.6. In Vitro Network Formation Assay

HUVECs (1 × 10^5^ cells/well) in different drug groups were seeded in a 96-well plate precoated with Matrigel (50 *μ*L/well, BD Biosciences, Bedford, MA 01730, USA) and transferred to a cell incubator with humidified 5% CO_2_ at 37°C for 24 h. The network structure was observed and imaged under an inverted microscope at 40x magnification. The angiogenic activities were evaluated by counting the branch points of the network structure formed, and the average numbers of branch points were measured from three random fields.

### 2.7. Transmission Electron Microscopy (TEM)

In observing the autophagosomes in HUVECs, HUVECs were harvested and fixed with 2.5% glutaraldehyde at 4°C for 2 h. The samples were then suspended in PBS containing 1% osmic acid at 4°C for 2 h. Following dehydration and embedding [18], ultrathin sections (60–70 nm) were prepared on uncoated copper grids using an Ultrotome and stained with uranyl acetate and lead citrate. Images were photographed using a TEM (H7650, Hitachi Limited, Japan).

### 2.8. Immunofluorescence Microscopy

Immunofluorescence was applied to assess the expression of autophagy protein, such as microtubule-associated protein 1A/1B-LC3-II, in the HUVEC samples from the normal, blank serum, H_2_O_2_, and H_2_O_2_ with LZD serum groups. Cells (2 × 10^4^) were seeded on 14 mm plates and cultivated for 24 h prior to intervention. Following three washes with PBS, the cells were then fixed with 4% paraformaldehyde at room temperature for half an hour and then washed three times with PBS. After permeabilization with 1% BSA/0.05% Triton X-100, the cells of the four groups were incubated with antibodies against LC3 (1 : 400, Cell Signaling Technology, USA) in a humidified container at 4°C for 12 h. After washing three times with PBS, the sections were incubated with Alexa Fluor® 488-conjugated AffiniPure Goat Anti-Rabbit IgG (H + L) secondary antibody (1 : 1,000, Cell Signaling Technology, USA) for 1 h at room temperature and then incubated with 0.0001% 4,6-diamidino-2-phe-nylindole (Sigma-Aldrich) for 10 min. After washing, the plates were examined under a fluorescence microscope [19].

### 2.9. Western Blot Analysis

HUVECs were cultured in 6-well plates with 1 × 10^6^ cells per plate. The cells were treated with different drug groups for 24 h and lysed with 80 *μ*l of sodium dodecyl sulfate (SDS) sample buffer (62.5 mM Tris-HCl [pH 6.8], 10% glycerol, 1% SDS, and 5% 2-mercapto-ethanol). The protein samples collected above were boiled at 100°C for 10 min before loading onto the acrylamide gel (12%) for SDS-polyacrylamide gel electrophoresis. A biotinylated ladder (Cell Signaling Technology, Danvers, MA, USA) was used as a size marker. After the proteins were separated by SDS-PAGE (Beyotime Biological Co., Ltd., China), they were transferred onto a nitrocellulose membrane at 90 V for 100 min. The membrane was blocked with a commercial protein blocking solution for 1 h after the blotting procedure, followed by washing three times with 1x Tris-buffered saline (TBS, 20 mM Tris-HCl, 136 mM NaCl, pH 7.6) containing 1% Tween-20 for 10 min. Then, the membrane was incubated overnight at 4°C with anti-LC3 (1 : 1,000, Cell Signaling Technology, USA), anti-Beclin-1 (1 : 1,000, Abcam, UK), and Anti-*β*-Actin (1 : 2,000, Santa Cruz Biotechnology, Inc., USA). On the next day, the membrane was washed three times with TBS for 15 min, followed by incubation with Goat Anti-Rabbit IgG (H + L) antibody (1 : 15,000, Cell Signaling Technology, USA) for 1 h at room temperature. After the membrane was washed three times with TBS for 15 min, the electrochemiluminescence detection system was used to analyze the blots.

## 3. Results

### 3.1. H_ 2_O_ 2_ Inhibits Cell Proliferation and LZD-Medicated Serum Significantly Promotes Injured HUVEC Proliferation

**Figure 1:**
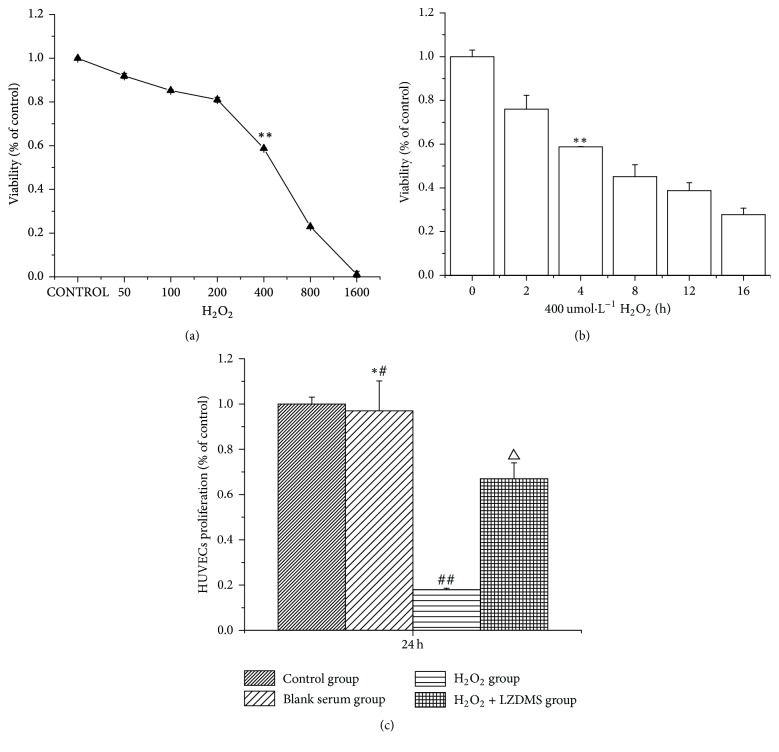
*Inhibitory effect of H*_*2*_ *O*_*2*_ *on HUVECs proliferation*. HUVECs, human umbilical vein endothelial cells; LZDMS, long-zhi decoction medicated serum. (a) Proliferation inhibition of HUVECs with different concentrations of H_2_O_2_ for 4 h. (b) Proliferation inhibition of HUVECs with 400 *μ*mol·L^−1^ of H_2_O_2_ at a series of time points. (c) The effect of each experimental group on HUVECs proliferation. Values are expressed as the mean ± standard error of the mean (*n* = 3). ^*∗∗*^*P* < 0.01 and ^*∗*#^*P* > 0.05, compared with the control group; ^##^*P* < 0.01, compared with the control group and blank serum group; ^△^*P* < 0.01, compared with the H_2_O_2_ group. LZDMS, long-zhi decoction medicated serum.

CCK8 assay was used to investigate cell proliferation. The optimal dosage and intervention time of H_2_O_2_ were determined by a preexperiment. The condition in which 400 *μ*mol/L of H_2_O_2_ was used to interfere with HUVECs for 4 h was suitable for the experiment (Figures 1(a)-1(b)) to determine whether LZD-medicated serum in HUVECs affected cell proliferation. The results showed that the cells with H_2_O_2_ were inhibited more significantly than those in the control group and that the cells in the H_2_O_2_ group treated with the LZD-medicated serum proliferated faster than those in the H_2_O_2_ group (Figure 1(c)).

### 3.2. Promotion of HUVEC Migration

**Figure 2:**
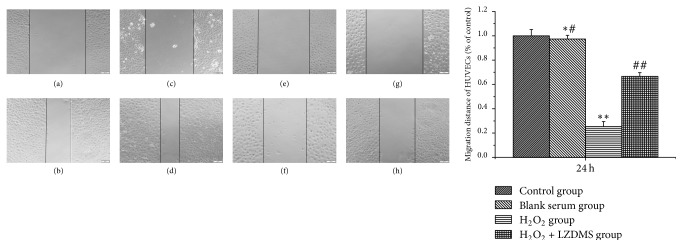
*Migration of HUVECs by wound scratch test*. HUVECs, human umbilical vein endothelial cells; LZDMS, long-zhi decoction medicated serum. (a, b) Control group: HUVECs scratch wound microscopic images at 0 h and 24 h. (c, d) The migrated distance of blank serum had no significant difference compared with the normal control group after 24 h. (e, f) H_2_O_2_ intervened HUVECs scratch wound at 0 h and 24 h. (g, h) The migrated distance of H_2_O_2_ with LZD-medicated serum treated HUVECs was much higher than H_2_O_2_, and LZD-medicated serum improved the migration of HUVECs. All experiments were performed in three replicates. ^*∗*#^*P* > 0.05 versus the control; ^*∗∗*^*P* < 0.01 versus the control; ^##^*P* < 0.05, versus the H_2_O_2_ group.

To determine whether the LZD-medicated serum was capable of promoting HUVEC migration, cells were treated with different drug groups in a 2D migration assay. The results showed that HUVEC migration was significantly promoted by the treatment of LZD-medicated serum. Relative to that of the control group, the average distance of migration of the H_2_O_2_ group cells to migrate was reduced to 25.33%. However, relative to that of the H_2_O_2_ group, the migration distance of cells treated with H_2_O_2_ and LZD-medicated serum increased to 66.67% (Figure 2).

### 3.3. Characterization of HUVECs and LZD-Enhanced Angiogenesis In Vitro

**Figure 3:**
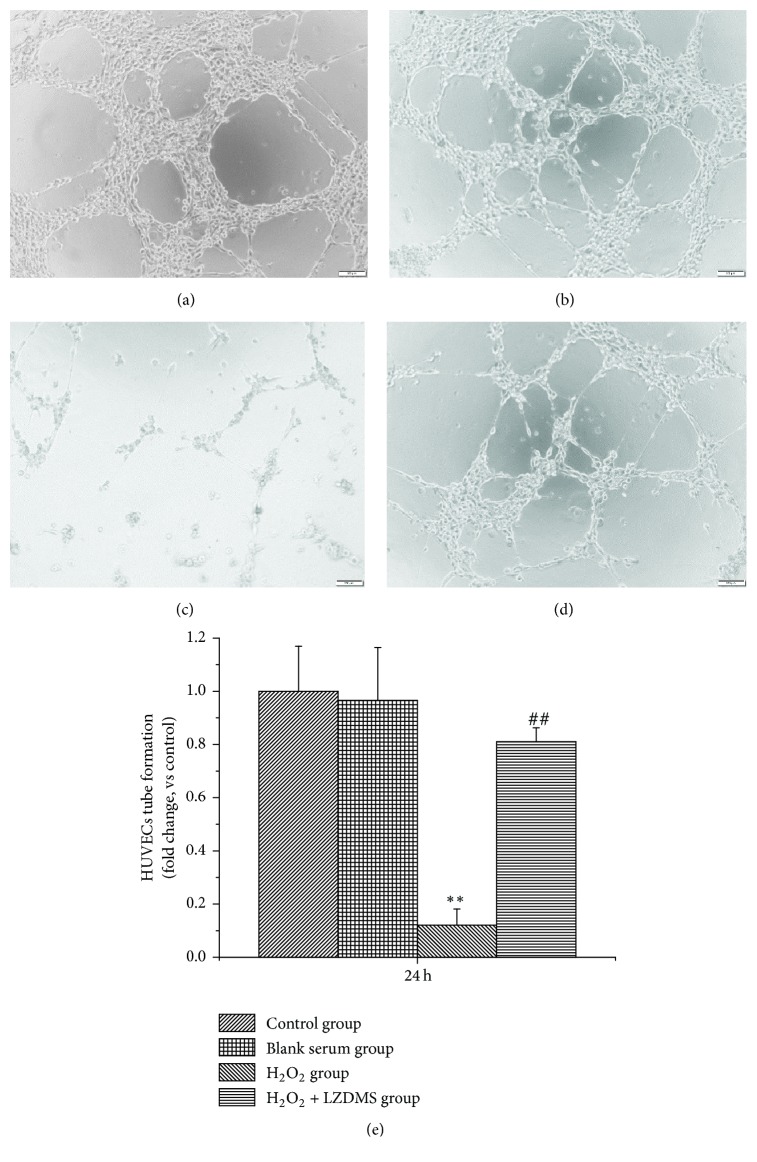
*Promoted effect of LZD-medicated serum on HUVECs tube formation*. HUVECs, human umbilical vein endothelial cells; LZDMS, long-zhi decoction medicated serum. The control group (a), HUVECs that were treated with blank serum (b), 400 *μ*moL·L^−1^ H_2_O_2_ (c), and 400 *μ*moL·L^−1^ H_2_O_2_ + LZD-medicated serum (d), respectively. ^*∗∗*^*P* < 0.05 versus the control, ^##^*P* < 0.05 versus H_2_O_2_ group. Data represent mean ± SD of three replicates.

Tube formation is a key step for angiogenesis. HUVECs formed tubular structures in the Matrigel, and the effect was examined 24 h after being seeded. Exposure to the angiogenesis inhibitor H_2_O_2_ resulted in the decrease in the capillary-like structures on the Matrigel. LZD treatment promoted the formation of tubular structures (Figure 3(d)). Fewer branch points were observed in the treated group with 400 *μ*mol/L H_2_O_2_ (Figure 3(c)) than in the control group (Figure 3(a)). No significant difference was found between the blank serum group (Figure 3(b)) and the control group. Quantitative measurements confirmed that exposure to LZD resulted in a significant increase in the mean number of branch points (Figure 3(e)). This result suggests that LZD may have a boosting effect on angiogenesis.

### 3.4. Production of Autophagosomes in HUVECs

**Figure 4:**
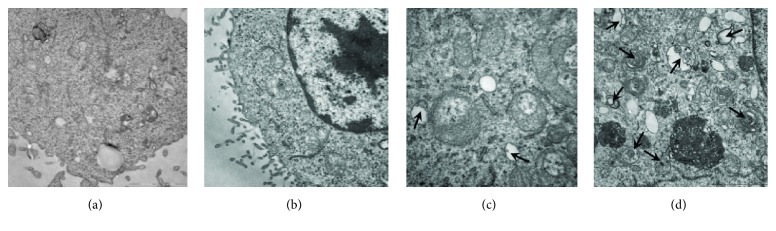
*The production of autophagosomes*. HUVECs, human umbilical vein endothelial cells; LZDMS, long-zhi decoction medicated serum. (a/b) Cells were treated with or without blank serum for 24 h, and autophagosomes were not detected by TEM. (c) Cells were treated with H_2_O_2_ (400 *μ*moL·L^−1^) for 24 h, and a small amount of autophagosomes was detected by TEM. (d) Cells were treated with H_2_O_2_ + LZD-medicated serum for 24 h, and more autophagosomes were detected by TEM.

To investigate whether H_2_O_2_ could induce autophagy and to observe the effect of autophagy level with an LZD-medicated serum on HUVECs, we used TEM to detect autophagosomes. Autophagic vacuoles are morphological hallmarks of autophagy [20]. As shown in Figure 4, the control group (Figure 4(a)) and the cells were treated with blank serum (Figure 4(b)) for 24 h, and autophagosomes were not detected by TEM. Autophagic vacuoles containing cellular material or membranous structures in medically treated cells were detected.

### 3.5. Immunofluorescence Detection of LC3-II Expression

**Figure 5:**
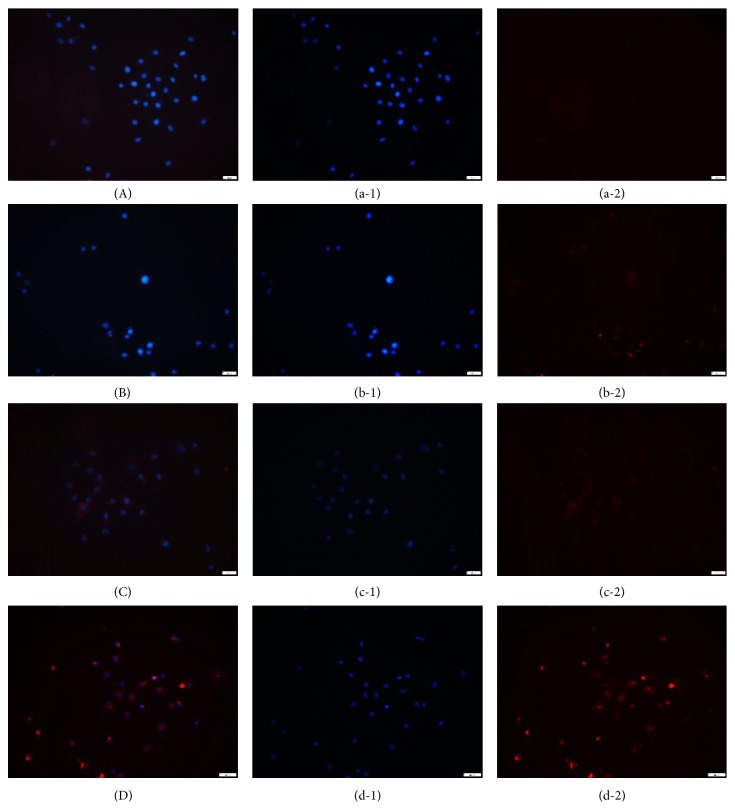
*Immunofluorescence of LC3-II expression*. HUVECs, human umbilical vein endothelial cells; LZDMS, long-zhi decoction medicated serum. (1) represents Nuclear staining; (2) represents cytoplasm staining; (A), (B), (C), and (D) represent a combination of nucleus and cytoplasmic staining. Immunofluorescence analysis showed extremely low LC3-II expression in the control group (A) and the blank serum group (B). The number of LC3-II positive HUVECs in the H_2_O_2_ group (C), and the intensity of LC3-II staining increased with the treatment of LZD-medicated serum (D). The results were obtained at 24 h.

LC3-II plays an important role in the formation of autophagic vacuoles. It is considered a marker for the activation of autophagosomes, and it is specifically localized in the double-layer membrane of autophagosomes. Thus, the immunofluorescence detection of LC3-II was used to determine the autophagic level. LC3-II expression was barely detectable in the control (Figure 5(A)) and blank serum groups (Figure 5(B)). In the experimental group, LC3-II was positive in HUVECs at 24 h (Figure 5(C)). The H_2_O_2_ pretreatment + LZD-medicated serum group showed a pattern of increased LC3-II expression at 24 h (Figure 5(D)) and exhibited a more intense staining pattern than the H_2_O_2_ group. However, the LZD-medicated serum was shown to promote autophagy in HUVECs.

### 3.6. Expression of LC3-II and Beclin-1 Protein by Western Blot

**Figure 6:**
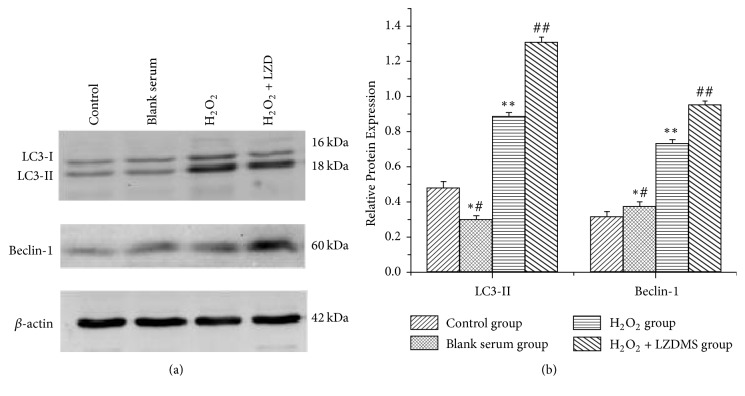
*Western blot detection of LC3 and Beclin-1 expression in HUVECs*. HUVECs, human umbilical vein endothelial cells; LZDMS, long-zhi decoction medicated serum. (a) Cells were exposed to the different group conditions for 24 h. The autophagy-related proteins were detected by Western blot, and *β*-actin was detected as a loading control. (b) Quantitative analysis of LC3-II and Beclin-1 protein relative to *β*-actin; data are presented as the mean ± standard deviation of 3 samples per group. ^*∗*#^*P* > 0.05 versus normal control group; ^*∗∗*^*P* < 0.05 versus normal control group; ^##^*P* < 0.05 versus H_2_O_2_ group.

To determine the effect of the LZD-medicated serum on cell autophagy of HUVECs, the expression of autophagy-related proteins LC3-II and Beclin-1 was examined using Western blot. The results showed that the conversion of LC3-I into LC3-II and Beclin-1 expressions increased after the LZD-medicated serum treatment compared with that of the H_2_O_2_ group (Figure 6(b)). No apparent expression of the autophagy-related protein was detected in the normal and blank serum groups (Figure 6(a)).

## 4. Discussion

IS is one of the major health issues in developing countries, and it leads to long-term morbidity and etiologies of mortality [21]. As previously mentioned, new vessel formation can provide therapeutic benefits in stroke management after IS. The underlying physiology of angiogenesis is complicated, as it involves the interaction of multiple signaling pathways and the expression of many growth factors, such as the vascular endothelial growth factor, transforming growth factors, platelet-derived growth factor, and basic fibroblast growth factor [22, 23]. Conventional angiogenesis studies usually focus on growth factors, but our previous experiments demonstrated that LZD could promote angiogenesis. In this study, experimental innovation was focused on the relevance between angiogenesis and cellular autophagy, and angiogenesis was explored from the perspective of cellular autophagy. The result shows that LZD-medicated serum promotes the migration, tube formation, and invasion of HUVECs accompanied by the upregulation of LC3-II and Beclin-1. This finding suggests that LZD may be a promising therapeutic drug to promote angiogenesis by improving cellular autophagy.

Autophagy occurs at a low basal level under physiological conditions in the body [24]. Autophagic elimination of injured mitochondria and endoplasmatic reticulum splinters could contribute to this protection because of the termination of apoptosis [25, 26]. In addition, many studies have shown that the process of angiogenesis is accompanied by an increase in autophagy [27]. The main novelty of this study demonstrated that autophagy is necessary for therapeutic angiogenesis [28]. In this study, we investigated the role of autophagy in the process of angiogenesis and deduced that H_2_O_2_ was activated by reactive oxygen species to injure HUVECs through autophagy [29]. Conversely, H_2_O_2_ was used to intervene in HUVECs, and autophagy models were established. H_2_O_2_ induces autophagy and increased LC3-II and Beclin-1 expression [30, 31]. Autophagy proteins and autophagosomes increased when using LZD to repair the damaged HUVECs.

Cell migration plays a vital role in the process of angiogenesis, and it is the basis for assessing angiogenesis at the cellular level [32]. This dynamic process of angiogenesis is induced by cytokines, chemical substances, and external stimuli [33]. This study presented various experimental groups of cell migration through the wound scratch test. After the comparison and analysis of the experimental results, the LZD-medicated serum seems to stimulate the vitality of damaged cells and enhance their migration rate in vitro, which is beneficial to the angiogenesis of HUVECs and may increase the migrated cell number in the ischemic area. We also utilize the in vitro Matrigel to simulate the formation of a vascular lumen [34, 35], although the process of Matrigel-induced morphological differentiation of HUVECs is unlikely to be the same as the formation of new vessels, which occurs during angiogenesis in vivo. The in vitro Matrigel system in combination with a numerical analyzer allows the easy and rapid quantitative determination of tube formation induced by the Matrigel [36]. The model can still be used to define other factors or molecular events affecting new blood vessel formation with endothelial cell [37].

LC3-II and Beclin-1 are considered key regulators of autophagy in mammalian cells. However, little is known about LC3-II and Beclin-1 expression in endothelial cells. LC3-II, which serves as an autophagosome-specific protein [38], is one of the most reliable markers of autophagosomes in mammals [39]. LC3-I exists in the cytoplasm, and LC3-II is a membrane-bound protein that attaches to autophagosomes and subsequently combines with lysosomes [40]. The amount of LC3-II reflects a large number of autophagosomes obtained through the process of LC3-I converting into LC3-II. Therefore, the induction and inhibition of autophagy could be monitored through the detection of LC3-II levels [41]. Cellular autophagic activity has been reported to occur in various types of cells [42]. Beclin-1 is strongly associated with the formation of autophagic membranes and may also be an important sign of autophagy [43]. Currently, Beclin-1 is usually studied in the field of tumors, including ovarian, breast, and prostate cancer [44]. In the present study, TEM demonstrated that autophagosomes increased with the LZD-medicated serum-induced new tube formation in HUVECs. In this process, Western blot analysis demonstrated that LC3-II and Beclin-1 expression increased, consistent with the result of fluorescence microscopy.

The debate on the effect of autophagy on the pathophysiology of cerebral ischemia remains unresolved. Mild-to-moderate autophagy has been proposed to encourage the survival of endothelium and that its overactivation promotes cellular death [45]. However, the current threshold for excessive autophagy is unclear [46]. Additionally, autophagic activities beneficial to vasculature cells may be involved in the protection of the integrity of angiogenesis. The effect of autophagy on the cell can be described as a double-edged sword [47]. H_2_O_2_ has been shown to induce autophagy above the basal level in HUVECs, as shown by a specific level of LC3-II and Beclin-1 [29]. Furthermore, the autophagy-related protein significantly improved when we added the LZD-medicated serum to protect the damaged cells and to promote HUVEC proliferation and tube formation. An increase in LC3-II may deter the completion of autophagy, and thus the autophagy shift can be assessed by measuring Beclin-1. The induction of an autophagic flux is usually accompanied by a high expression of LC3-II, but not of LC3-I, and increased amounts of Beclin-1.

## 5. Conclusion

In summary, the present result showed that the LZD-medicated serum promoted the proliferation, migration, and tube formation of HUVECs accompanied by the upregulation of LC3-II and Beclin-1. This finding suggests that LZD may be a promising therapeutic drug to improve brain function in IS. The autophagy of LZD-medicated serum-induced HUVECs may be involved in the process of new tube formation. The result further reveals that cellular autophagy has the potential to be a therapeutic strategy for IS and is a possible way to promote angiogenesis.
